# Early anterior cingulate involvement is seen in presymptomatic *MAPT* P301L mutation carriers

**DOI:** 10.1186/s13195-021-00777-9

**Published:** 2021-02-10

**Authors:** Mica T. M. Clarke, Frédéric St-Onge, Jean-Mathieu Beauregard, Martina Bocchetta, Emily Todd, David M. Cash, Jonathan D. Rohrer, Robert Laforce

**Affiliations:** 1 Dementia Research Centre, Department of Neurodegenerative Disease, UCL Queen Square Institute of Neurology, London, UK; 2 Centre de Recherche du CHU de Québec, Université Laval, Québec, QC Canada; 3 Clinique Interdisciplinaire de Mémoire (CIME), Département des Sciences Neurologiques, CHU de Québec-Université Laval, Québec, QC Canada; 4 Faculté de Médecine, Département de médecine, Université Laval, Québec, QC Canada; 5 CHU de Québec, Québec, QC Canada; 6 Universite Laval, Québec, QC Canada

**Keywords:** PET imaging, Glucose metabolism, Presymptomatic, Frontotemporal dementia

## Abstract

**Background:**

PET imaging of glucose metabolism has revealed presymptomatic abnormalities in genetic FTD but has not been explored in *MAPT* P301L mutation carriers. This study aimed to explore the patterns of presymptomatic hypometabolism and atrophy in *MAPT* P301L mutation carriers.

**Methods:**

Eighteen asymptomatic members from five families with a P301L *MAPT* mutation were recruited to the study, six mutation carriers, and twelve mutation-negative controls. All participants underwent standard behavioural and cognitive assessment as well as [^18^F]FDG-PET and 3D T1-weighted MRI brain scans. Regional standardised uptake value ratios (SUVR) for the PET scan and volumes calculated from an automated segmentation for the MRI were obtained and compared between the mutation carrier and control groups.

**Results:**

The mean (standard deviation) estimated years from symptom onset was 12.5 (3.6) in the mutation carrier group with a range of 7 to 18 years. No differences in cognition were seen between the groups, and all mutation carriers had a global CDR plus NACC FTLD of 0. Significant reduction in [^18^F] FDG uptake in the anterior cingulate was seen in mutation carriers (mean 1.25 [standard deviation 0.07]) compared to controls (1.36 [0.09]). A similar significant reduction was also seen in grey matter volume in the anterior cingulate in mutation carriers (0.60% [0.06%]) compared to controls (0.68% [0.08%]). No other group differences were seen in other regions.

**Conclusions:**

Anterior cingulate hypometabolism and atrophy are both apparent presymptomatically in a cohort of P301L *MAPT* mutation carriers. Such a specific marker may prove to be helpful in stratification of presymptomatic mutation carriers in future trials.

**Supplementary Information:**

The online version contains supplementary material available at 10.1186/s13195-021-00777-9.

## Background

Frontotemporal dementia (FTD) describes a clinically, genetically and pathologically heterogenous group of diseases characterised by degeneration of the frontal and temporal cortices [30]. Approximately a third of all FTD is genetic, with the first described cause being mutations in the microtubule-associated protein tau (*MAPT*) gene in 1998 [13, 22, 27]. Over 70 pathogenic mutations have since been discovered, with the most common being the P301L mutation in exon 10 [19].

In recent years, several studies have identified presymptomatic changes in genetic FTD using a range of neuroimaging techniques, although the majority of these have focused on structural, functional or perfusion magnetic resonance imaging (MRI) [6, 9–11, 21, 24]. Studies of positron emission tomography (PET) imaging have been more limited, and mainly focused on [^18^F]-fluorodeoxyglucose (FDG-PET), a measure of glucose metabolism in vivo, where hypometabolism is thought to reflect neuronal dysfunction. In limited studies so far, FDG-PET has revealed presymptomatic abnormalities in other genetic causes of FTD [2, 7, 8, 14], but not in P301L *MAPT* mutation carriers.

In this study, we aimed to investigate whether presymptomatic neuronal dysfunction is present in P301L *MAPT* mutation carriers as measured by FDG-PET, and whether patterns of hypometabolism differed from patterns of atrophy.

## Methods

### Participants

Eighteen asymptomatic participants were recruited from five families with an autosomal dominant P301L mutation in the *MAPT* gene through the Quebec City (Canada) site of the Genetic Frontotemporal dementia Initiative (GENFI) study. All participants underwent genetic screening with 6 found to be carriers of the mutation, and 12 found to be mutation-negative, and therefore used as controls in this study. There were no significant differences in age (*p* = 0.98) or sex (*p* = 0.32) between groups. Participants and investigators were blinded to individual genetic statuses. The study was approved by the CHU de Québec-Université Laval (Québec City, Canada) research ethics board. Written informed consent was obtained from all participants before any study-related procedures.

### Clinical assessment

Each participant underwent a standardised assessment (Table 1) including the Mini-Mental State Examination (MMSE), Montreal Cognitive Assessment (MoCA), Frontal Assessment Battery (FAB) and the CDR® Dementia Staging Instrument with National Alzheimer Coordinating Centre Frontotemporal Lobar Degeneration component (CDR® plus NACC FTLD) which generates both global and sum of boxes scores. Estimated years from symptom onset were calculated for each participant as the current age taken away from the mean age at onset within the participant’s family [19] (Table 1).

**Table 1:** Demographics, behavioural and cognitive features in P301L *MAPT* mutation carriers (*n* = 6) and controls (*n* = 12)

	Mutation carriers, mean (SD)	Controls, mean (SD)	***U***	***p***
**Age (years)**	44.8 (6.3)	46.1 (7.2)	35.50	0.98
**Sex (male:female)**	1:5	6:6	N/A	0.32
**Estimated years from symptom onset**	12.5 (3.6), range: 7–18 years	9.5 (9.1), range: − 5.5–29 years	24.50	0.30
**Years of education**	16.3 (2.3)	14.4 (2.2)	21.50	0.17
**CDR with NACC FTLD sum of boxes**	0.0 (0.0)	0.1 (0.2)	30.00	0.53
**Mini-Mental State Examination (/30)**	29.8 (0.4)	29.8 (0.5)	33.00	> 0.99
**Montreal Cognitive Assessment (/30)**	28.5 (1.8)	28.3 (1.7)	33.50	0.83
**Frontal Assessment Battery (/18)**	17.8 (0.4)	17.2 (0.9)	20.00	0.16

### Neuroimaging

Participants underwent an FDG-PET scan acquired on a Siemens Biograph 6 PET/CT scanner (Siemens Medical Solutions, Erlangen, Germany). After the intravenous injection of 185–370 MBq of [^18^F] FDG, an uptake period of 30–45 min was observed. A low-dose, non-contrast CT of the head was acquired for attenuation correction and anatomical correlation, followed by a 15-min PET acquisition in 3D mode, which was then reconstructed using ordered subset expectation maximisation with a point-spread function (Siemens HD-PET) with a 128 × 128 matrix (2.7 × 2.7 × 3.0 mm^3^ voxel).

All participants also underwent T1-weighted MRI on a 3T Siemens MAGNETOM Skyra (Siemens Healthcare, Erlangen, Germany) within 6 months of their PET-CT scan visit. Regions of interest (ROI) were defined on the co-registered T1-weighted MR image using Geodesic Information Flow (GIF), a previously described brain parcellation methodology [5, 24]. This generated eight lobar regions (frontal, temporal, parietal, occipital, insula, anterior cingulate, middle cingulate and posterior cingulate) and five subcortical regions (amygdala, hippocampus, caudate, putamen and thalamus). Frontal, temporal, parietal and occipital lobes comprised bilateral grey and white matter GIF labels whilst insula and cingulate regions comprised grey matter only. All segmentations underwent quality control: this revealed poor segmentation of caudate and putamen structures for some participants and so these were not included in standardised uptake value ratio (SUVR) or volumetric analyses (two carriers and four controls for the caudate, one carrier and three controls for the putamen).

Reconstructed PET data frames were averaged together. T1-weighted MR images were coregistered with the corresponding FDG-PET data using SPM 12 (v12.1) in Matlab R2017a and the PET images were transformed (and upsampled) into the MR space. Partial volume correction was applied to upsampled PET SUVR data using the iterative Yang method (6.8 mm kernel, 10 iterations). SUVR of the PET data was computed using the cerebellum region (as defined by the GIF parcellation). Using the MRI-defined regions of interest, SUVR values were produced for each of the lobar and subcortical regions specified above. Both scans were then transformed using NiftyReg to the MNI152 standard-space template included in FSL.

Volumetric data was expressed as a percentage of total intracranial volume (TIV), measured using SPM 12 as a combination of grey matter, white matter and cerebrospinal fluid segmentations.

### Statistical analysis

Statistical analyses were performed in RStudio Version 1.2.5033, GraphPad Prism Versions 8.4.3 and 9.0.0 and SPSS Version 27. Shapiro-Wilk normality tests revealed data were not normally distributed for all variables, including demographic, cognitive and imaging data, so non-parametric Mann-Whitney *U* tests were used to compare measures between mutation carrier and control groups. For significant group comparisons for the imaging measures, receiver operating characteristic (ROC) curve analyses were performed to assess the diagnostic capacity of each marker. ROC curve analyses for SUVR and volume biomarker measures combined were also performed by first performing a logistic regression model using carrier/control as the outcome variable and SUVR and volume measures as the covariates to calculate the predicted probabilities, then using the predicted probabilities as the input variable for a combined ROC curve.

## Results

Demographic, cognitive and behavioural features of the sample are presented in Table 1. There were no significant differences in age, sex distribution, estimated years from symptom onset or years of education between groups. The mean (standard deviation) estimated years from onset was 12.5 (3.6) in the mutation carrier group with a range of 7 to 18 years. Group comparisons of cognitive and behavioural measures revealed no significant differences between the groups in the MMSE, MoCA or FAB. All mutation carriers and ten out of the twelve controls had a CDR NACC-FTLD sum of boxes (and therefore global score also) of 0. Two of the controls had a CDR NACC-FTLD sum of boxes (and therefore global score also) of 0.5.

### Regional [^18^F] FDG uptake

**Fig. 1:**
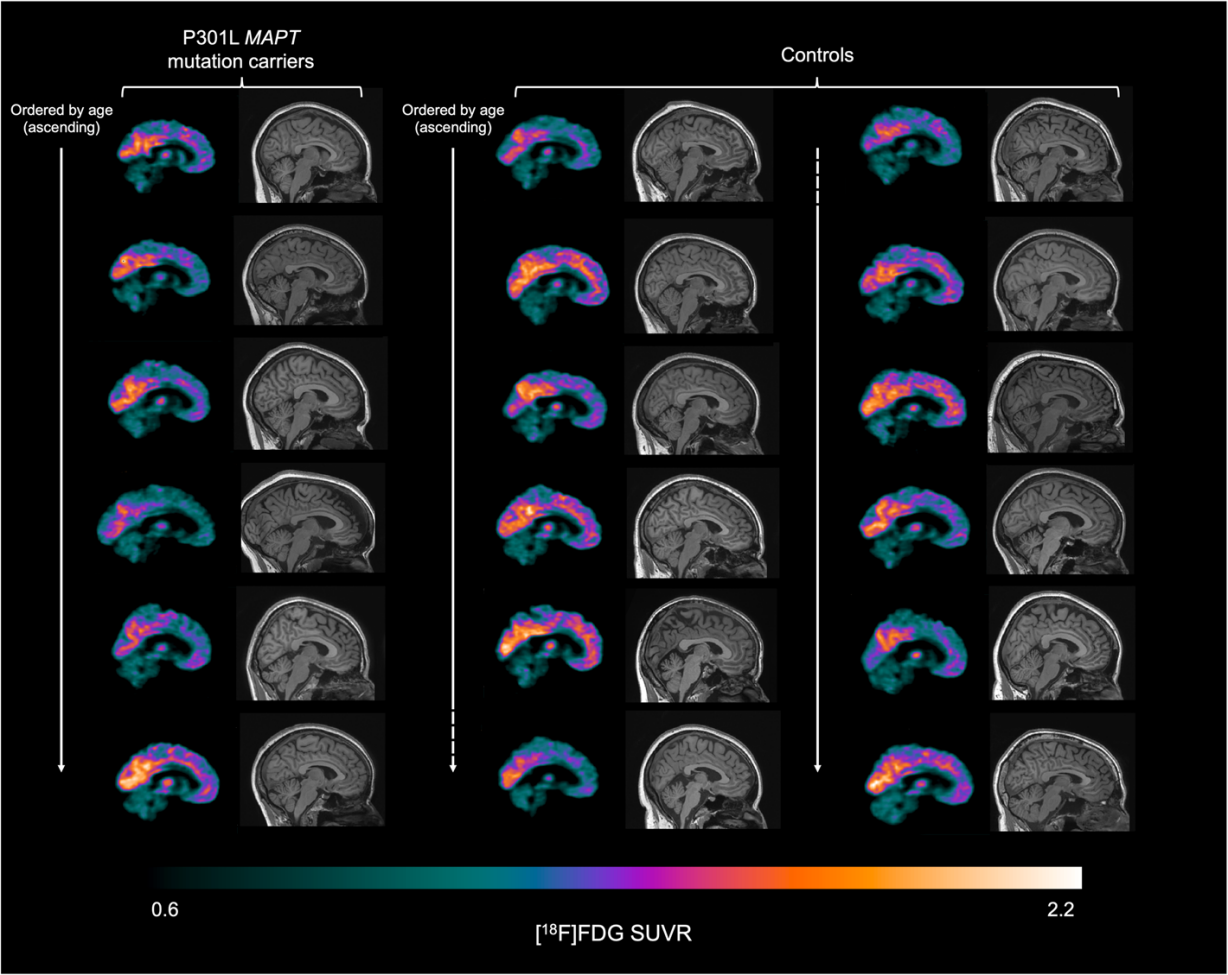
Sagittal slices illustrating the [^18^F] FDG SUVR in P301L *MAPT* mutation carriers (left column, *n* = 6) and controls (middle and right columns, *n* = 12). The same slice is presented for all participants. Slices are presented in order of ascending age

**Fig. 2:**
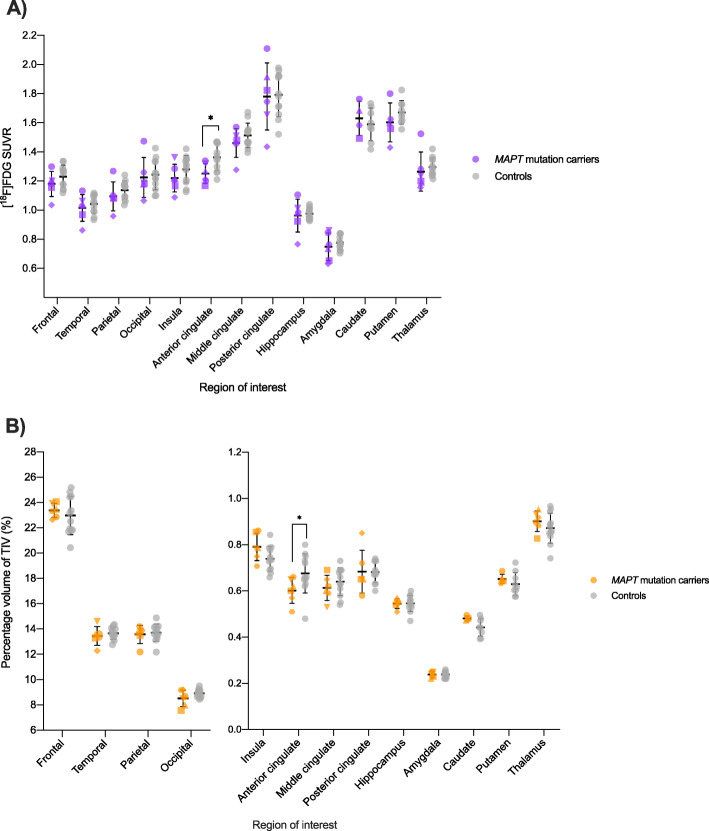
**a** Individual regional SUVR of [^18^F] FDG in *MAPT* mutation carriers and controls with group means and standard deviations. Purple datapoints symbolise the carrier group. **b** Individual regional volumes as a percentage of TIV in *MAPT* mutation carriers and controls with group means and standard deviations. Orange datapoints symbolise mutation carriers. Individual carriers are represented by individual shapes: circle, square, upwards-pointing triangle, downwards-pointing triangle, diamond and hexagon. Grey datapoints symbolise the control group. *p ≤ 0.05

Group comparisons of regional SUVR in *MAPT* mutation carriers and controls revealed a significant reduction in [^18^F] FDG uptake in the anterior cingulate in P301L *MAPT* mutation carriers (mean 1.25 [standard deviation 0.07]) compared to controls (1.36 [0.09]; *U* = 12.00, *p* = 0.02) (Figs. 1 and 2a, Table 2). There were no significant differences in uptake in any other cortical or subcortical regions (Fig. 2a, Table 2). A ROC curve analysis revealed an area under the curve (AUC) of 0.83 for anterior cingulate SUVR (standard error = 0.10, 95% confidence interval = 0.64 to 1.00, *p* = 0.03) (Supplementary Figure 1A).

**Table 2:** Group comparisons of regional SUVR and brain volumes in P301L *MAPT* mutation carriers (*n* = 6) and controls (*n* = 12). Significant results in bold and italics

Region of interest	FDG-PET imaging: regional SUVR				MR imaging: % of total intracranial volume			
	Mutation carriers, mean (SD)	Controls, mean (SD)	***U***	***p***	Mutation carriers, mean (SD)	Controls, mean (SD)	***U***	***p***
**Frontal**	1.18 (0.09)	1.23 (0.08)	27.00	0.44	23.38 (0.56)	22.97 (1.50)	29.00	0.55
**Temporal**	1.02 (0.09)	1.04 (0.06)	31.00	0.68	13.45 (0.75)	13.66 (0.48)	30.00	0.60
**Parietal**	1.09 (0.10)	1.14 (0.06)	26.00	0.38	13.57 (0.72)	13.71 (0.68)	35.00	0.96
**Occipital**	1.22 (0.14)	1.24 (0.10)	31.00	0.68	8.51 (0.64)	8.91 (0.31)	24.50	0.30
**Insula**	1.22 (0.09)	1.28 (0.09)	24.00	0.29	0.79 (0.06)	0.74 (0.05)	18.00	0.10
**Anterior cingulate**	***1.25 (0.07)***	***1.36 (0.09)***	***12.00***	***0.02***	***0.60 (0.06)***	***0.68 (0.08)***	***14.50***	***0.04***
**Middle cingulate**	1.46 (0.10)	1.51 (0.08)	30.00	0.62	0.61 (0.05)	0.64 (0.06)	25.50	0.34
**Posterior cingulate**	1.78 (0.23)	1.79 (0.15)	34.00	0.89	0.68 (0.09)	0.68 (0.05)	29.00	0.54
**Hippocampus**	0.96 (0.11)	0.98 (0.04)	35.00	0.96	0.55 (0.02)	0.55 (0.03)	35.00	0.95
**Amygdala**	0.75 (0.10)	0.78 (0.05)	32.00	0.75	0.24 (0.01)	0.24 (0.01)	35.00	0.91
**Caudate**	1.63 (0.12)	1.59 (0.11)	12.00	0.57	0.48 (0.01)	0.44 (0.04)	6.00	0.11
**Putamen**	1.60 (0.13)	1.67 (0.08)	13.00	0.24	0.65 (0.02)	0.63 (0.05)	14.00	0.30
**Thalamus**	1.26 (0.13)	1.30 (0.06)	19.00	0.12	0.90 (0.04)	0.87 (0.06)	26.00	0.38

### Regional volume

Group comparisons of regional volumes also revealed a significant reduction in grey matter volume in the anterior cingulate in P301L *MAPT* mutation carriers (0.60% [0.06%]) compared to controls (0.68% [0.08%]; *U* = 14.50, *p* = 0.04) (Fig. 2b). No other group differences were identified (Fig. 2b, Table 2). A ROC curve analysis revealed an AUC of 0.80 for anterior cingulate volume (standard error = 0.11, 95% confidence interval = 0.59 to 1.00, *p* = 0.04) (Supplementary Figure 1B).

A ROC curve analysis for combined anterior cingulate SUVR and volume measures revealed an AUC of 0.85 (standard error = 0.09, 95% confidence interval = 0.66 to 1.00, *p* = 0.02) (Supplementary Figure 1C).

## Discussion

The present study demonstrated reduced uptake of [^18^F] FDG and reduced grey matter volume in the anterior cingulate in P301L *MAPT* mutation carriers. The mean estimated years from symptom onset in this group was 12.5 years, with the nearest participant to onset being 7 years away, suggesting very early presymptomatic involvement of the anterior cingulate in genetic FTD caused by this mutation. Reduced uptake or brain volume was not found in any other lobar or subcortical region of interest.

Few studies have explored FDG-PET in presymptomatic FTD. A study of FDG-PET in presymptomatic *GRN* carriers also revealed significant reductions in uptake in the anterior cingulate but only in the right hemisphere [14], whilst a study of presymptomatic *C9orf72* repeat expansion carriers showed clusters of hypometabolism in frontal, temporal and insular cortices plus subcortical regions but no changes in the anterior cingulate [7]. Two studies have revealed temporal hypometabolism in presymptomatic *MAPT* mutation carriers, but with different variants to this study (N279K: [2]; 10 + 3: [8]): no studies have previously explored FDG-PET in P301L *MAPT* mutation carriers.

Patterns of atrophy in genetic FTD measured by MRI reveal common anterior cingulate involvement in symptomatic *MAPT*, *GRN* and *C9orf72* mutation carriers [6]. However, anterior cingulate change has not been widely reported presymptomatically. Atrophy of the cingulate cortex was not detected until just prior to expected symptom onset in a combined cohort of presymptomatic carriers of FTD-causing mutations [24]. In a separate analysis of *MAPT* mutation carriers, the earliest detected change was in the hippocampus and amygdala, followed by temporal and insular cortices. However, this group contained a mixed cohort of different *MAPT* mutations, with few P301L mutation carriers. One prior study has identified distinct patterns of atrophy depending on the specific *MAPT* mutation [31] highlighting the importance of investigating the anatomical changes in individual mutations.

P301L mutations tend to cause a more rapidly progressive disease than other *MAPT* mutations once symptomatic [19] and whilst the same mutation can give rise to multiple phenotypes, most symptomatic individuals exhibit behavioural disturbances and personality change [3, 12, 15, 18]. Early involvement of the anterior cingulate may explain why P301L *MAPT* mutation carriers typically present with symptoms of behavioural variant FTD. The cingulate cortex can be divided into functionally distinct regions including anterior, middle and posterior cingulate. Broadly, the anterior cingulate has been deemed ‘executive’ in function [29]. It is thought to modulate attention and executive functions by influencing response selection, and lesions of the anterior cingulate have produced inattention and apathy [4]. The anterior cingulate is also thought to play a critical role in social cognition via contextual integration and evaluating the behaviour of others [1, 16]. Apathy, executive dysfunction and social cognitive impairment are all core symptoms for the diagnosis of bvFTD [23, 33].

Early anterior cingulate involvement may be attributed to its unique constitution of von Economo neurons (VENs) which exhibit selective vulnerability in FTD [25]. VENs are thought to enable humans to act quickly and intuitively in social situations, and provide fast communication with the anterior insula within the salience network, a group of regions controlling social and emotional responses [26, 28]. One post-mortem study found the anterior cingulate was one of the regions most affected by tau aggregation in *MAPT*-associated FTD, with disproportionate tau aggregation in VENs [17]. Furthermore, a study of the salience network using resting state functional MRI reported a trend to reduced anterior cingulate connectivity in presymptomatic *MAPT* mutation carriers [32].

The identification of early anterior cingulate involvement via two independent measures increases the reliability of our findings in this study. Of note, partial volume correction was applied in our analyses (therefore mitigating the effect of atrophy on the resulting SUVR signal), suggesting that anterior cingulate hypometabolism cannot only be attributed to the neuronal loss seen on MRI, and that FDG-PET may provide complementary information about neuronal dysfunction in presymptomatic P301L *MAPT* mutation carriers. Certainly, prior investigation of FDG-PET in presymptomatic Alzheimer's disease (AD) has illustrated widespread hypometabolism in areas associated with AD pathology in the absence of widespread atrophy, suggesting neuronal dysfunction precedes neuronal loss [20]. However, in identifying parallel changes in FDG-PET and volumetric data in the anterior cingulate, it is difficult to be clear from this cross-sectional study alone whether hypometabolic change appeared before or concurrently with regional atrophy in P301L *MAPT* mutation carriers. Nonetheless, ROC curve analyses suggested good diagnostic ability of both anterior cingulate hypometabolism and volume in distinguishing presymptomatic P301L *MAPT* mutation carriers from controls, with a marginally greater AUC for the FDG-PET signal, and an even greater AUC when combining the two outcome measures, suggesting the use of both biomarkers is superior to either one individually. Future studies should focus on identifying P301L *MAPT* mutation carriers early on in the disease process prior to neuronal loss, when FDG-PET alone may be abnormal, with longitudinal follow-up to assess progression.

### Limitations

The study is limited by the small sample size and requires replication in a larger cohort. Poor segmentation of subcortical structures required some cases to be removed from specific ROI group comparisons, an inherent limitation of automated segmentation methods.

## Conclusions

In summary, this study of P301L *MAPT* mutation carriers shows early anterior cingulate involvement measured by both FDG-PET and MRI. Such early and specific changes may well be important in stratifying presymptomatic participants in the context of clinical trials, but future studies need to replicate these findings and understand the longitudinal changes over time in this population.

## Supplementary Information

**Additional file 1: Supplementary Figure 1.** Receiver operating characteristic (ROC) curves illustrate the capacity for A) anterior cingulate SUVR of [^18^F] FDG, B) anterior cingulate volume expressed as a percentage of TIV and C) combined anterior cingulate SUVR and volume biomarkers to distinguish P301L *MAPT* mutation carriers from controls.

## Data Availability

The datasets used and/or analysed during the current study are available from the corresponding author on reasonable request.

## Abbreviations

FTD: Frontotemporal dementia
MAPT: Microtubule-associated protein tau
MRI: Magnetic resonance imaging
PET: Positron emission tomography
FDG-PET: [^18^F] fluorodeoxyglucose PET
GENFI: Genetic Frontotemporal dementia Initiative
MMSE: Mini-Mental State Examination
MoCA: Montreal Cognitive Assessment
FAB: Frontal Assessment Battery
CDR plus NACC FTLD: CDR plus National Alzheimer Coordinating Centre Frontotemporal Lobar Degeneration component
ROI: Regions of interest
GIF: Geodesic Information Flow
SUVR: Standardised uptake value ratio
TIV: Total intracranial volume
VENs: Von Economo neurons
