# Supplementary figures and images for: Early anterior cingulate involvement is seen in presymptomatic MAPT P301L mutation carriers
Source: Alzheimers Res Ther. 2021 Feb 10;13:42. doi: 10.1186/s13195-021-00777-9 (PMC7876816; doi:10.1186/s13195-021-00777-9)

A) SUVR

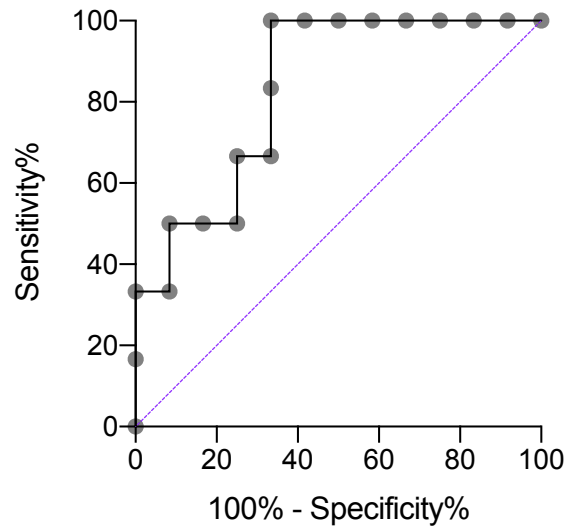

B) Volume

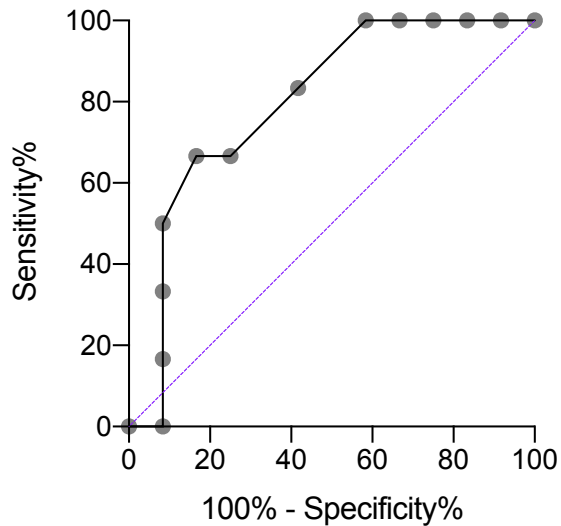

C) SUVR &amp; volume

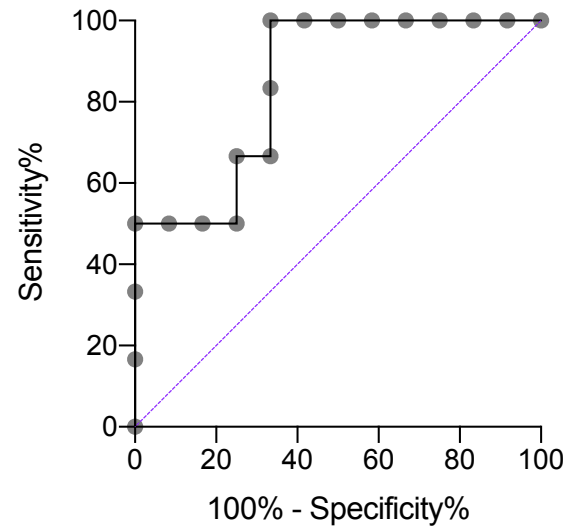

Supplement: Supplementary file 1 — Additional file 1: Supplementary Figure 1. Receiver operating characteristic (ROC) curves illustrate the capacity for A) anterior cingulate SUVR of [18F] FDG, B) anterior cingulate volume expressed as a percentage of TIV and C) combined anterior cingulate SUVR and volume biomarkers to distinguish P301L MAPT mutation carriers from controls. [file 13195_2021_777_MOESM1_ESM.pdf]
